# Polyhydroyxalkanoate Synthase Fusions as a Strategy for Oriented Enzyme Immobilisation

**DOI:** 10.3390/molecules19068629

**Published:** 2014-06-24

**Authors:** David O. Hooks, Mark Venning-Slater, Jinping Du, Bernd H. A. Rehm

**Affiliations:** 1 Institute of Fundamental Sciences, Massey University, Private Bag 11222, Palmerston North 4442, New Zealand; E-Mails: d.o.hooks@massey.ac.nz (D.O.H.); m.venningslater@massey.ac.nz (M.V.-S.); j.du@massey.ac.nz (J.D.); 2 MacDiarmid Institute for Advanced Materials and Nanotechnology, Kelburn Parade, Wellington 6140, New Zealand

**Keywords:** *Ralstonia eutropha*, polyhydroxyalkanoate synthase, enzyme immobilisation

## Abstract

Polyhydroxyalkanoate (PHA) is a carbon storage polymer produced by certain bacteria in unbalanced nutrient conditions. The PHA forms spherical inclusions surrounded by granule associate proteins including the PHA synthase (PhaC). Recently, the intracellular formation of PHA granules with covalently attached synthase from *Ralstonia eutropha* has been exploited as a novel strategy for oriented enzyme immobilisation. Fusing the enzyme of interest to PHA synthase results in a bifunctional protein able to produce PHA granules and immobilise the active enzyme of choice to the granule surface. Functionalised PHA granules can be isolated from the bacterial hosts, such as *Escherichia coli*, and maintain enzymatic activity in a wide variety of assay conditions. This approach to oriented enzyme immobilisation has produced higher enzyme activities and product levels than non-oriented immobilisation techniques such as protein inclusion based particles. Here, enzyme immobilisation via PHA synthase fusion is reviewed in terms of the genetic designs, the choices of enzymes, the control of enzyme orientations, as well as their current and potential applications.

## 1. Oriented Enzyme Immobilisation

Enzymes can be viewed as industrial catalysts and have applications ranging from fine chemical synthesis to environmental bioremediation [1,2]. In general, enzymes have high activity under chemically mild conditions (low temperature, atmospheric pressure, near-neutral pH) and high specificity for their substrates. Despite these advantages, industrial use of enzymes can be hindered by lack of stability, the challenge of separation from product, and difficulty recycling the soluble enzyme. For multimeric enzymes the stability problem is even more pronounced as dissociation of the subunits at low concentrations is the first step in deactivation of the enzyme and leads to additional contamination of the final product. Immobilisation of enzymes overcomes these drawbacks. Immobilising an enzyme enhances performance under harsh conditions (temperature, pH, *etc*.) and increases stability [3]. Immobilisation also allows simple separation of the catalyst from the product and facilitates re-use. Three broad categories of enzyme immobilisation exist: attachment to a solid support, encapsulation within a carrier, and carrier-free cross-linking [4]. In some techniques, the activity of the enzyme can be enhanced and its selectivity altered. If performed with care, immobilisation can also prevent subunit dissolution and keep the local concentration of enzyme high [5].

In addition to the increase in stability and recyclability, immobilisation provides the opportunity to co-localise multiple enzymes creating a synthetic multienzyme complex (MEC) that mimics structures found in nature and allows for more complex, multistep reaction *in vitro* [6]. Further, oriented immobilisation has been considered important for higher catalytic activity, optimal electron exchange between the redox enzymes and support material, and activation of hydrophobic active sites for hydrophobic substrates [7]. In the case of redox enzymes, the enzyme active site needs to be in close proximity to the carrier so the transfer of electrons can occur. In other cases, such as the immobilisation of subtilisin, the active site may need to be fully exposed to the solute to allow efficient access by the substrate [8]. Using particular attachment sites can alter an enzymes properties including enhanced stability [3]. Finally, oriented enzyme immobilisation is also thought to be crucial for the optimal function and miniaturisation of biosensors [7]. This review covers the advances made in a particular *in vivo* enzyme immobilisation technique using the natural properties of bacterial polyhydroxyalkanoate (PHA) inclusion formation to generate oriented display of enzymes attached to a solid support.

## 2. Polyhydroxyalkanoate Biobeads

PHAs, also referred to as bioplastics or biopolyesters, are naturally occurring polyesters composed of (*R*)-3-hydroxy fatty acids. Many bacteria and some archaea are able to produce PHAs under carbon-excess and nitrogen-limiting conditions [9]. These polyesters are stored as water-insoluble inclusions inside the cells and serve as energy and carbon storage polymers [10,11,12].

Formation of the most common short chain-length PHAs, such as polyhydroxybutyrate, requires three key enzymes. The β-ketothiolase (PhaA) catalyses the condensation of acetyl-CoA, acetoacetyl-CoA reductase (PhaB) reduces the condensation product acetoacetyl-CoA into PHA precursor molecule (*R*)-3-hydroxybutyryl-CoA, and the PHA synthase (PhaC) polymerises the precursor molecule into PHA [13,14]. The PHA biosynthesis pathway from the PHA inclusion model organism *Ralstonia eutropha* has been successfully transferred to and established in recombinant bacteria like Gram-negative *Escherichia coli,* as well as Gram-positive organisms such as *Corynebacterium glutamicum* and *Lactococcus lactis* [15,16,17]. In these recombinant systems, PhaA and PhaB are produced as soluble proteins. The PhaC is under the control of an inducible T7 promoter and the expressed enzyme remains covalently attached to the growing PHA chain.

As the PHA synthase (PhaC) remains covalently attached to the polyester core of the inclusions, it is considered as an important tag for anchoring target proteins on the surface of PHAs and to produce functionalised biobeads. This is achieved by first constructing fusions of the PhaC to proteins of interest. Production of these fusion proteins in recombinant bacteria (either natural or engineered PHA producers) leads to the formation of functionalised PHA biobeads. A significant advantage to this approach is the elimination of a chemical attachment step of the free enzyme to a support material. This crosslinking step is often expensive or difficult and results in a random orientation of enzymes to the support material surface. In contrast, the PhaC fusions anchor the protein of interest in a defined orientation to the biopolyester surface *in vivo* meaning the protein of interest is being immobilised prior to isolation. Other ways to get oriented enzyme display have been reviewed, including site-directed mutagenesis [18].

## 3. *In Vivo* Immobilisation and Surface Display

In natural producers of PHB, phasins (PhaP) can comprise up to 5% of the intracellular proteins making it the most abundant granule-associated protein [19]. The potential for PhaP to be used as the anchor protein for recombinant enzyme immobilisation and display was demonstrated by the fusion of β-galactosidase to PhaP via a self-cleaving intein [20]. PHA biobeads were separated from lysed cells by gradient centrifugation and the β-galactosidase was cleaved using a DTT-containing solution. The purified β-galactosidase had a specific activity of 53 U/μmol, comparable to other purification methods [20]. Additionally, recombinant human tissue plasminogen activator (rPA) was fused to PhaP with a thrombin cleavage site as the linker [21]. As rPA is a serine protease with multiple disulfide bonds, recombinant expression is difficult. In the fibrin degradation assay, rPA was found to be active both when attached to the biobeads and after cleavage into solution with thrombin [21].

PhaC can be divided into Classes I to IV based on quaternary structure and composition of the PHA synthesised. Not only Class I but also Class II PhaC has been demonstrated capable of immobilising enzymes on PHA biobeads. Both Class I and Class II PhaC consist of only one subunit. However Class I PhaC produces short-chain-length PHA, while Class II PhaC yields medium-chain-length PHA [10].

In 2006 β-galactosidase was successfully immobilised on PHA beads using a PHA-negative mutant of *Pseudomonas aeruginosa* and by fusing this enzyme N-terminally to Class II PHA synthase from *P. aeruginosa* [22]. Further studies switched to an engineered *E. coli* PHA producing system using N- and/or C- terminal fusions to Class I PhaC from *R. eutropha* in order to immobilise a series of technical enzymes [23,24,25,26].

A variety of enzymes ranging from oxidoreductase (NemA), hydrolase (for example, β-galactosidase, LacZ [EC 3.2.1.23]; α-amylase, BLA [EC 3.2.1.1]; and organophosphohydrolase, OpdA [EC 3.1.8.1]), lyase (*N*-acetylneuraminic acid aldolase, NanA [EC 4.1.3.3]), to isomerase (*N-*acetylglucosamine 2-epimerase, Slr1975 [EC 5.1.3.8]) were successfully immobilised to PHA biobeads [23,24,25,26]. These enzymes differed in quaternary structures from monomer (BLA), dimer (OpdA, Slr1975) to tetramer (NanA, LacZ), and ranged in monomer size from about 33 kDa (NanA) to 116 kDa (LacZ) [27,28,29,30,31]. In addition, two of the immobilised enzymes (BLA and OpdA) are secreted in their original hosts. Enzymes originated from a variety of hosts such as Gram-positive bacterium *Bacillus licheniformis* (BLA); as well as Gram-negative bacteria *E. coli* (LacZ, NemA, NanA), *Agrobacterium radiobacter* (OpdA) and the cyanobacterium *Synechocystis* sp. (Slr1975)*.*

Immobilisation of enzymes to PHA biobeads by fusion to either PhaP or PhaC has been shown to be viable. PhaP attaches to the PHA biobead through hydrophobic interactions whereas PhaC is stabilised by a covalent attachment to the PHA polymer itself. Additionally, the use of PhaP as an anchoring protein requires an extra gene which is strictly unnecessary for the recombinant production of PHA biobeads. Elimination of PhaP allows for PhaC to coat the outer surface of the PHA biobeads. Possibly due to its weaker interaction with the PHA biobead, PhaP is used when isolation of soluble enzyme is desired [20,21]. For enzyme immobilisation, PhaC has been seen as the anchoring protein of choice, especially if harsh reaction conditions and multiple recycles are required [24,25,26].

## 4.*In Vitro* Immobilisation and Surface Display

In addition to the * in vivo* approach, PHA biobeads can also be produced *in vitro* [32,33]. This method involves first expressing and purifying the PhaC-fusion protein from a recombinant host then PHB nanoparticles (diameter 200 nm) are formed from pure PHB. Finally, the PhaC-fusion protein, PHB nanoparticles, and the PhaC substrate 3-hydroxybutyryl-coenzyme A (3-HB-CoA) are mixed together. PhaC polymerises the 3-HB-CoA forming a fusion protein-PHB hybrid molecule. The growing hydrophobic PHB chain is pushed towards the hydrophobic PHB nanoparticle with the fusion protein facing the aqueous environment. So far, this approach has only been used to immobilise the targeting peptide RGD4C [32] and green fluorescent protein (GFP) [33] but the same principle could apply to enzyme immobilisation in the future. The RGD4C tagged biobeads were shown to be targeted to MDA-MB 231 breast cancer cells *in vitro* and able to deliver the lipophilic dye Nile Red as a model drug [32]. The advantages of an *in vitro* approach to PHA biobeads are control over biobead purity as well as size which can be modulated by the amount of 3-HB-CoA added to the emulsion. The disadvantages are the increase in complexity and production costs when compared to the one-step *in vivo* immobilisation of fusion protein.

## 5. Orientation of Biobead Immobilised Enzymes

The orientation of an enzyme crosslinked to a support material is critical for its functionality/activity *i.e.*, the ability to bind and convert a substrate. For example, structural analysis of a lipase from *Thermomyces lanuginosus* (TLL) showed that the enzyme possesses an alpha-helical surface loop lid (a common structure of lipases) that governs access of reaction media to the hydrophobic active site [34,35,36]. TLL, like most lipases, is activated at the oil/water interface which induces the lid to open providing access to the active site of the enzyme. Immobilisation of TLL to a hydrophobic support by utilising this interfacial activation, orientates the enzyme so that it is stabilised in its open conformation and leads to hyperactive levels of activity [37,38].

**Figure 1:**
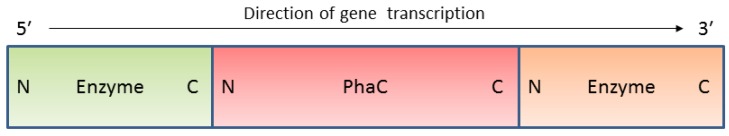
Potential sites of enzyme attachment utilising gene fusions to PHA synthase. N,N-terminus; C,C-terminus.

As described above, the enzymes fused to PhaC are displayed on the surface of PHA beads. PhaC dictates a homogenous orientation of its fusion partner at the surface of the beads which, if properly engineered, provides maximum interaction with substrate, hence leading to high specific activity of the immobilised enzyme. Additionally, the non-porous nature of the PHA beads avoids the problem of substrate diffusion by enabling convective interactions. Due to the co-translation of enzyme-PhaC fusions, enzymes are orientated in one of two directions, depending on whether the fusion occurs at the N or C-terminus (Figure 1). For example, a variant of a thermostable α-amylase from *B. licheniformis* has been fused by its C-terminus to PhaC and lead to the successful production and isolation of PHA beads that exhibit enzyme activity [23]. Furthermore, in two separate studies, a chromium (VI) reductase (NemA) from *E. coli* and an organophosphate hydrolase from *A. radiobacter* (OpdA) have been fused to PhaC via their N-termini, respectively, and have also resulted in the isolation of enzymatically active PHA beads [24,26]. However, there is evidence that orientation via fusion at either terminus can affect enzyme activity. PHA beads were produced that displayed an *N*-acetylneuraminic acid aldolase (NanA) from *E. coli* that was fused by either its N or C terminus [25]. Under optimal conditions it was shown that fusion protein which had NanA fused at its C-terminus to PhaC (NanA-PhaC) produced beads that exhibited superior NanA activity compared to beads formed from PhaC-NanA. This study also demonstrated the possibility of fusing two enzymes to the PhaC termini simultaneously using NanA and an *N*-acetylglucosamine 2-epimerase (Slr1975) from *Synechocystis* sp. strain PCC 6803. Although beads displaying single enzymes exhibited higher activity, functional dual-enzyme beads could still be obtained, highlighting the potential for immobilising enzymes of the same pathway in close proximity.

## 6. Quaternary Structures of Immobilised Enzymes

Enzymes used in industry encompass a range of quaternary structures, and the correct conformation of these structures is critical in the display of enzyme activity. Therefore, an enzyme immobilisation system must have a strong bond between enzyme and support while allowing flexibility for the enzyme to fold correctly, *i.e.*, to form its functional quaternary structure. Various enzymes have been immobilised using the PHA immobilisation system and have retained activity, demonstrating the versatility of this system.

A modified thermostable α-amylase (BLA) from *B. licheniformis* was immobilised and displayed on PHA beads [23]. BLA is a monomeric enzyme with a molecular mass of approximately 55 kDa [29]. Interestingly, the BLA-PHA beads were thermostable retaining activity after incubation at 85 °C for 5 h [23]. The OpdA quaternary structure can be deduced from an extensively studied homologue from *Brevundimonas diminuta* (a.k.a *Pseudomonas diminuta*) which has been shown to be dimeric [28]. NanA has been shown to be homotetrameric where each subunit consists of an α/β-barrel domain and three α-helices extending from the C-terminus [31]. Although comparisons with free NanA enzymes were not within the scope of this study, NanA-PhaC and PhaC-NanA beads demonstrated aldolase activity with NanA-PhaC exhibiting superior activity at 590 U/g dry bead weight [25]. In addition, NanA was also fused to PhaC in conjunction with the dimeric enzyme *N*-acetylglucosamine 2-epimerase (Slr1975) from *Synechocystis* sp. strain PCC 6803 to yield beads displaying both enzymes [25]. The structure of a homologous epimerase has been resolved [30]. Interestingly, only NanA-PhaC-Slr1975 beads displayed activity, suggesting limits on the placement of enzyme functions within the PhaC fusion protein. However, Slr1975-PhaC-NanA beads did form, demonstrating the robustness of the PhaC enzyme in exhibiting its own enzyme activity and bead forming ability.

PHA beads have been shown to be capable of displaying active enzymes with varying quaternary structures, and also in different combinations. Being able to fuse enzyme functions to both the N and C-termini of PhaC, as single and dual enzyme functions, demonstrates the versatility and potential of PHA beads in enzyme applications.

## 7. The Initial Proof of Concept of PHA Synthase Mediated Enzyme Immobilisation

As mentioned above, the first proof for PHA synthase mediated enzyme immobilisation was obtained in 2006 [22]. β-galactosidase-displaying PHA beads were produced by fusing β-galactosidase from *E. coli* to the N-terminus of Class II PhaC from *P. aeruginosa*.

The PHA-negative mutant of *P. aeruginosa* PAO1 was complemented by plasmid pBBR1JO5-lacZphaC1 containing the LacZ-PhaC fusion protein encoding gene under *lac* promoter control. Cells were cultivated under conditions favouring PHA bead accumulation. Upon bead isolation, the β-galactosidase was demonstrated to be covalently immobilised on the bead surface and showed an activity of 68,000 MU on average, with a *K_m_* of 630 µM and a *V*_max_ of 17.6 nmol/min using orthonitrophenyl-β-d-galactopyranoside as substrate. This result shed lights on protein engineering of PHA synthase as a platform technology for efficient covalent enzyme immobilisation.

## 8. Current Applications

PHA beads have been used to immobilise a variety of enzymes that can be used for a range of applications from food production to the synthesis of fine chemicals. The successful display of a thermostable α-amylase (BLA) could be used in a wide variety of industries that require starch liquefaction including food processing, detergent manufacture, paper processing, and textiles [39,40,41]. Immobilised enzymes have a potential use in the bioremediation of toxic chemicals which would otherwise persist in the environment. Hexavalent chromium is a water-soluble toxin generated by a range of industries including pigment production, leather tanning, wood preservation, stainless steel manufacture, and nuclear technology [24,42]. PHA beads displaying pollutant detoxifying or degrading enzymes will likely enhance the residence time of the respective enzyme in the polluted environment while being fully biodegraded over time. In combination with either *Bacillus subtilis* glucose dehydrogenase or *Candida boidinii* formate dehydrogenase as a cofactor generating partner, PHA beads displaying NemA were able to transform toxic, water-soluble chromium (VI) to relatively non-toxic, water-insoluble chromium (III) [24]. Another bioremediation target is the build-up of certain pesticides. Organophosphate pesticides are highly toxic and intentionally used in farming, particularly in the developing world, and account for approximately 200,000 deaths per year and are considered as a significant global health problem by the World Health Organisation [43,44]. Due to such use there is the potential of contamination to water supplies. PHA beads displaying OpdA were able to effectively degrade the organophosphate insecticide coumaphos present in undiluted wool scour in less than two hours [26]. Finally, PHA beads displaying enzymes have potential use in fine chemical synthesis. The NanA and Slr1975 enzymes can catalyse a reaction pathway involved in the synthesis of *N*-acetylneuraminic acid (Neu5Ac), a precursor of an antiviral used as a neuraminidase inhibitor for the treatment of influenza virus infections [45,46,47]. Both single enzyme and double-enzyme beads were successfully employed in the production of this fine chemical, and represent an alternative to chemical synthesis [25]. The successful display of active enzymes that exhibit a range of quaternary structure and activity illustrates the versatility of the PHA bead enzyme immobilisation system for research and industry.

## 9. Performance of PHA Bead Immobilized Enzymes

The initial fusion of LacZ to PhaC possessed immobilised LacZ with a binding affinity of 630 µM [22] which was high when compared with the method of attaching LacZ covalently to gold-coated devices [48]. Immobilisation of the α-amylase to PHA beads resulted in Michaelis-Menten kinetics with V_max_ at 506 mU/mg bead protein and a K_m_ of 5 µM [23] which is consistent with free α-amylase at 9.6 µM but less than measured for α-amylase attached to cellulose beads of 44 µM [40]. After three cycles of reaction the immobilised α-amylase retained 78% of its initial activity.

In the case of OpdA immobilisation, the K_m_ of covalently attached PhaC-OpdA protein was found to be 250 µM, about 1.6 times higher than free OpdA at 160 µM [26]. The apparent k_cat_ for attached PhaC-OpdA was 139 s^−1^; 16 times lower than for free OpdA at 2300 s^−1^. These values result in a second order rate constant (k_cat_/K_m_) of 5.5 × 10^5^ M^−1^s^−1^ for attached PhaC-OpdA and 1.4 × 10^7^ M^−1^s^−1^ for free OpdA. The thermal properties were also assessed with attached PhaC-OpdA having slightly more thermal stability than free OpdA. An increase of 1.58 °C in apparent melting temperature and a slow decline in activity from 35–80 °C was seen in attached PhaC-OpdA compared to a rapid decline in activity for free OpdA at 55–70 °C. Both can perform in the demanding reaction environment of wool scour. Long-term stability was assessed in tap water at 25 °C with little loss in activity or difference between the attached and free OpdA observed after 11 days. After 5 months storage, 15% of the attached OpdA activity remained [26].

Immobilisation of NemA to PHA beads resulted in a substantial increase in the K_m_ for chromium VI to 95 µM compared to the free NemA at 1.7 µM. However, the K_m_ for NADH was essentially identical: 49 µM for attached NemA compared to 45 µM for free NemA [24]. Even though the immobilisation reduced the ability of NemA to transform chromium VI, the data indicated that it remained an efficient chromium VI reductase. Stability of the immobilised NemA was high, after nine months at 4 °C no reduction in enzyme activity was detectable [24].

Activity of Slr1975 fusion protein immobilised to PHA beads was measured at 1.8 U/mg and 0.58 U/mg depending on the arrangement of the fusion protein [25]. These values are lower than the previously reported activities of immobilised GlcNAc-2 epimerases which range from 3.4–29 U/mg protein [49,50]. During the same study, NanA was also immobilised to the PHA beads. The activity of NanA fusion protein was measured at 43 U/mg and 82 U/mg [49,50], again depending on the arrangement of the fusion protein. These values compared favourably with the previously reported activities of immobilised NanA between 2.5–36 U/mg protein. Moreover, an alternative GFP fusion protein particle approach for immobilisation of NanA resulted in specific NanA activity of 76 mU/mg [51].

In general, the effect of immobilising an enzyme to the PHA beads is an increase in the K_m_ when compared to free enzyme. However, when compared to other immobilisation methods, the PHA system performs to approximately the same level in regard to the measured properties with some variation depending on the enzyme of interest. The direct comparison of specific enzymes immobilised to PHA beads and immobilised to GFP fusion protein particles is particularly relevant because of the similarity in fusion protein construction. In all three cases, NanA, α-amylase, and OpdA the performance under the same reaction conditions is less for the GFP fusion protein particles when compared to the equivalent PHA bead [51]. This is likely to be due to differences in the way the different particles form and the resultant orientation and surface exposure of the respective enzyme. In the PHA beads the enzyme is displayed on the bead surface which allows for active site access by the substrate. In the GFP fusion particles, the enzyme may be buried in a random orientation and with limited substrate access to the enzyme.

## 10. Potential Applications

**Figure 2:**
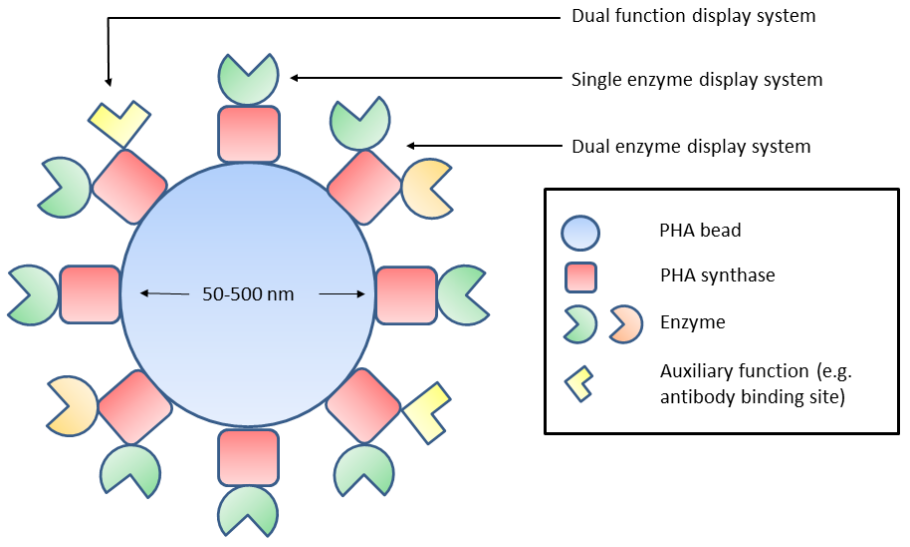
Potential applications of the PHA bead display system.

The PHA synthase enzyme immobilization system is amenable to functional fusions on both termini simultaneously [52], even with two different enzymes [25], or a protein functionality of choice (Figure 2). This creates an opportunity for a multistep catalysis reaction or biomolecular interaction to be established. In nature, many enzymes are known to operate in multi-enzyme complexes (MEC), for example tryptophan synthase, or the pyruvate dehydrogenase complex. Display systems which allow the immobilisation of two or more enzymes can be useful as a model of biological enzyme complexes. MECs have high local concentration of intermediates, lessening reaction inefficiencies caused by diffusion.

This is especially valuable for highly reactive intermediates which degrade quickly. Of critical importance in a dual enzyme immobilisation system is the relative positioning of the enzymes and their level of interaction. In most dual immobilisation systems, steric hindrance prevents compact display. However, in the PHA system, the enzymes are brought together by the initial fusion, dimerization of PhaC, and formation of a PHA bead with a surface display of the fusion protein. Further, there is some evidence suggesting the PhaC dual fusion partners are in close proximity [25,52]. The potential of the PHA immobilisation system to enhance dual enzyme display could be further explored with the widely used model glucose oxidase/horse radish peroxidase (GOx/HRP) glucose detection system. Certain dual enzyme display approaches have been shown to enhance intermediate hydrogen peroxide conversion up to 250% compared with the equivalent amount of free enzyme in solution [53]. Other systems could include a main enzyme and a secondary helper enzyme which enhances enzymatic activity by regeneration of cofactors. An example of fine chemical synthesis by a dual enzyme system is d-hydantoinase/d-carboamylase (d-case) which mediates the formation of the optically pure antibiotic precursor, p-hydroxyphenylglycine (d-pHPG), from a racemic mixture of hydroxyphenylhydantoin [54]. In commercial preparation of d-pHPG, a chemical process is used for the second step due to the high oxidative sensitivity and low stability of d-case. A dual enzyme system immobilised to chitin had increased pH stability and activity at low temperatures [54]. Finally, the enzyme pair could work in tandem to produce a greater result than either could produce alone, such as the industrially useful enzyme laccase combined with HRP. This combination allows the breakdown of lignin to occur to a greater extent than each enzyme separately [55].

## 11. Discussion

The recent studies on the PHA biobead enzyme display technology have revealed the flexibility of the system. It is able to successfully express enzymes with different functional roles, varying quaternary structure, and potential applications. The K_m_ is often, but not always, higher for the immobilised enzyme than the free enzyme meaning lower substrate binding affinity for the attached enzyme. In the case of immobilised OpdA, k_cat_ was also substantially reduced [26]. However, it is expected that immobilised enzymes would have weaker substrate affinities when compared to their free counterparts in solution. One of the main advantages of enzyme immobilisation is the ability to recycle the enzyme over multiple uses. The α-amylase displaying beads were able to be recycled over three reactions while the NanA and Slr1975 displaying beads were able to be used over five cycles each [23,25].

Additional research has included studying the function of enzymes displayed on PHA beads in comparison to enzymes immobilised to GFP fusion protein particles [51]. Samples were standardised based on the amount of fusion protein per reaction, and then subjected to a variety of treatments followed by the respective activity assay. Beads and particles displaying BLA, NanA, and OpdA were assessed. In all cases enzyme-bearing PHA beads catalysed higher rates of reaction, as well as achieved higher levels of product formation [51]. This difference in activity is possibly due to differences in the way in which PHA beads and GFP particles form, and the resultant display of the enzymes. As explained above, the PhaC is responsible for the formation of PHA beads, and remains covalently attached at the bead surface [12]. Therefore, any enzyme that is fused to PhaC is also displayed on the bead surface, and has ready access to substrate. Although no model for GFP particle formation has been proposed, evidence suggests that they are similar to inclusion bodies consisting of GFP fusion proteins that contain a fusion of GFP, an inactive variant of PhaC (PhaC(C319A)), and the relevant enzyme fused at the C-terminus. The inactive PhaC was also found to be replaceable by different polypeptides serving as linker between engineered GFP and the fused target protein. Inclusion bodies are protein aggregates that have been commonly observed in bacteria that have been engineered to overproduce recombinant proteins [56]. They form through the aggregation of partially misfolded proteins that have been overproduced, which form proto-aggregates that aggregate further to form mature inclusion bodies [57,58]. Furthermore, structural analysis of inclusion bodies has identified native-like secondary structure of the constituent proteins and amyloid-like cross-molecular β-sheet formations indicating an ordered process of formation [56,59]. Although inclusion bodies exhibit an ordered structure the constituent proteins are displayed throughout the aggregate. In terms of enzyme-bearing GFP particles, this means there is little control on the placement and orientation of the enzyme fusions. As the assays were standardised on the amount of fusion protein, most of the fusion protein used in the GFP particle reaction was below the surface of the particle separated from substrate. Furthermore, it is also possible that the enzyme portion of the surface fusion proteins are still buried below the surface of the particles, or in an orientation that restricts movement, therefore, resulting in decreased activity. Moreover, if the formation of GFP particles abides by the current model of inclusion body formation not all of the fusion protein of the GFP particles will be correctly folded [56,58]. Therefore, only a fraction of the fusion protein in GFP particles will consist of active enzyme of which only a fraction will have access to substrate. Observing decreased enzyme activity of GFP particles in comparison to PHA beads indicates a favourable comparison to inclusion bodies, and that some enzyme molecules of the GFP fusion proteins are not optimally placed. Interestingly, GFP particles have been shown to function comparably to PHA beads, and even to a higher extent, in terms of IgG binding capacity when fused to the IgG binding domain ZZ derived from protein A[60]. This could be due to differences in particle density, shape, surface area, the IgG binding sites in comparison to enzymes, and the ability of the ZZ-domain to rapidly fold after translation. However, despite this, enzyme activity comparisons based on fusion protein quantity highlights the importance of providing favourable conditions for enzyme protein folding, and for controlled enzyme placement and orientation.

## 12. Outlook

The PHA beads immobilisation method was proven to be versatile as a variety of functionally and structurally diverse enzymes could be actively immobilised. Compared to other immobilisation methods the enzymatic properties of the PHA beads were not always superior. However, very little was undertaken to optimize the immobilisation strategy, *i.e.*, improve the design of the PhaC fusion protein. For example LacZ had a higher K_m_ and Slr1975 had a lower activity but NanA had a higher activity than other NanA immobilisation techniques [22]. Direct experimental comparison of oriented PHA biobeads to GFP fusion particles revealed that enzymes immobilised to PHA beads consistently reached higher product formation rates [51]. This effect was observed for enzymes active as monomers, dimers, and tetramers indicating the advantages and flexibility of the PHA biobead system. The production levels of 1.5 g per litre of bacterial culture for at least some enzyme biobeads indicate a high level of volumetric productivity [24]. Attention to design of the fusion protein is needed as enzymes which may be functional when expressed on one terminus of PhaC are sometimes not functional when fused to the other [25]. Future research in this field will include double enzyme fusions especially in high-value product lines such as biofuels, flavanoids, and optically pure pharmaceuticals.

## References

[1] Fernández-Fernández M., Sanromán M.Á., Moldes D. (2013). Recent developments and applications of immobilized laccase. Biotechnol. Adv..

[2] Muñoz Solano D., Hoyos P., Hernáiz M.J., Alcántara A.R., Sánchez-Montero J.M. (2012). Industrial biotransformations in the synthesis of building blocks leading to enantiopure drugs. Bioresour. Technol..

[3] Mateo C., Palomo J.M., Fernandez-Lorente G., Guisan J.M., Fernandez-Lafuente R. (2007). Improvement of enzyme activity, stability and selectivity via immobilization techniques. Enzyme Microb. Technol..

[4] Sheldon R.A., van Pelt S. (2013). Enzyme immobilisation in biocatalysis: Why, what and how. Chem. Soc. Rev..

[5] Fernandez-Lafuente R. (2009). Stabilization of multimeric enzymes: Strategies to prevent subunit dissociation. Enzyme Microb. Technol..

[6] Jia F., Narasimhan B., Mallapragada S. (2013). Materials-based strategies for multi-enzyme immobilization and co-localization: A review. Biotechnol. Bioeng..

[7] Steen Redeker E., Ta D.T., Cortens D., Billen B., Guedens W., Adriaensens P. (2013). Protein engineering for directed immobilization. Bioconjug. Chem..

[8] Huang W., Wang J., Bhattacharyya D., Bachas L.G. (1997). Improving the activity of immobilized subtilisin by site-specific attachment to surfaces. Anal. Chem..

[9] Tan G.-Y., Chen C.-L., Li L., Ge L., Wang L., Razaad I., Li Y., Zhao L., Mo Y., Wang J.-Y. (2014). Start a Research on Biopolymer Polyhydroxyalkanoate (PHA): A Review. Polymers (Basel).

[10] Rehm B.H.A. (2003). Polyester synthases: Natural catalysts for plastics. Biochem. J..

[11] Rehm B.H.A. (2006). Genetics and biochemistry of polyhydroxyalkanoate granule self-assembly: The key role of polyester synthases. Biotechnol. Lett..

[12] Grage K., Jahns A.C., Parlane N., Palanisamy R., Rasiah I.A., Atwood J.A., Rehm B.H.A. (2009). Bacterial polyhydroxyalkanoate granules: Biogenesis, structure, and potential use as nano-/micro-beads in biotechnological and biomedical applications. Biomacromolecules.

[13] Normi Y.M., Hiraishi T., Taguchi S., Abe H., Sudesh K., Najimudin N., Doi Y. (2005). Characterization and properties of G4X mutants of Ralstonia eutropha PHA synthase for poly(3-hydroxybutyrate) biosynthesis in Escherichia coli. Macromol. Biosci..

[14] Satoh Y., Tajima K., Tannai H., Munekata M. (2003). Enzyme-catalyzed poly(3-hydroxybutyrate) synthesis from acetate with CoA recycling and NADPH regeneration *in vitro*. J. Biosci. Bioeng..

[15] Jo S.-J., Maeda M., Ooi T., Taguchi S. (2006). Production system for biodegradable polyester polyhydroxybutyrate by Corynebacterium glutamicum. J. Biosci. Bioeng..

[16] Mifune J., Grage K., Rehm B.H.A. (2009). Production of functionalized biopolyester granules by recombinant Lactococcus lactis. Appl. Environ. Microbiol..

[17] Valappil S.P., Boccaccini A.R., Bucke C., Roy I. (2007). Polyhydroxyalkanoates in Gram-positive bacteria: Insights from the genera Bacillus and Streptomyces. Antonie Van Leeuwenhoek.

[18] Hernandez K., Fernandez-Lafuente R. (2011). Control of protein immobilization: Coupling immobilization and site-directed mutagenesis to improve biocatalyst or biosensor performance. Enzyme Microb. Technol..

[19] Steinbuchel A., Aerts K., Babel W., Follner C., Liebergesell M., Madkour M.H., Mayer F., Pieper-Furst U., Pries A., Valentin H.E. (1995). Considerations on the structure and biochemistry of bacterial polyhydroxyalkanoic acid inclusions. Can. J. Microbiol..

[20] Barnard G.C., McCool J.D., Wood D.W., Gerngross T.U. (2005). Integrated recombinant protein expression and purification platform based on Ralstonia eutropha. Appl. Environ. Microbiol..

[21] Geng Y., Wang S., Qi Q. (2010). Expression of active recombinant human tissue-type plasminogen activator by using *in vivo* polyhydroxybutyrate granule display. Appl. Environ. Microbiol..

[22] Peters V., Rehm B.H.A. (2006). *In vivo* enzyme immobilization by use of engineered polyhydroxyalkanoate synthase. Appl. Environ. Microbiol..

[23] Rasiah I.A., Rehm B.H.A. (2009). One-step production of immobilized alpha-amylase in recombinant Escherichia coli. Appl. Environ. Microbiol..

[24] Robins K.J., Hooks D.O., Rehm B.H.A., Ackerley D.F. (2013). Escherichia coli NemA is an efficient chromate reductase that can be biologically immobilized to provide a cell free system for remediation of hexavalent chromium. PLoS One.

[25] Hooks D.O., Blatchford P.A., Rehm B.H.A. (2013). Bioengineering of bacterial polymer inclusions catalyzing the synthesis of N-acetylneuraminic acid. Appl. Environ. Microbiol..

[26] Blatchford P.A., Scott C., French N., Rehm B.H.A. (2012). Immobilization of organophosphohydrolase OpdA from Agrobacterium radiobacter by overproduction at the surface of polyester inclusions inside engineered Escherichia coli. Biotechnol. Bioeng..

[27] Juers D.H., Jacobson R.H., Wigley D., Zhang X.J., Huber R.E., Tronrud D.E., Matthews B.W. (2000). High resolution refinement of beta-galactosidase in a new crystal form reveals multiple metal-binding sites and provides a structural basis for alpha-complementation. Protein Sci..

[28] Benning M.M., Shim H., Raushel F.M., Holden H.M. (2001). High resolution X-ray structures of different metal-substituted forms of phosphotriesterase from Pseudomonas diminuta. Biochemistry.

[29] Hwang K.Y., Song H.K., Chang C., Lee J., Lee S.Y., Kim K.K., Choe S., Sweet R.M., Suh S.W. (1997). Crystal structure of thermostable alpha-amylase from Bacillus licheniformis refined at 1.7 A resolution. Mol. Cells.

[30] Lee Y.-C., Wu H.-M., Chang Y.-N., Wang W.-C., Hsu W.-H. (2007). The central cavity from the (alpha/alpha)6 barrel structure of Anabaena sp. CH1 N-acetyl-D-glucosamine 2-epimerase contains two key histidine residues for reversible conversion. J. Mol. Biol..

[31] Izard T., Lawrence M.C., Malby R.L., Lilley G.G., Colman P.M. (1994). The three-dimensional structure of N-acetylneuraminate lyase from Escherichia coli. Structure.

[32] Kim H.-N., Lee J., Kim H.-Y., Kim Y.-R. (2009). Enzymatic synthesis of a drug delivery system based on polyhydroxyalkanoate-protein block copolymers. Chem. Commun. (Camb.).

[33] Lee J., Jung S.-G., Park C.-S., Kim H.-Y., Batt C.A., Kim Y.-R. (2011). Tumor-specific hybrid polyhydroxybutyrate nanoparticle: Surface modification of nanoparticle by enzymatically synthesized functional block copolymer. Bioorg. Med. Chem. Lett..

[34] Derewenda U., Swenson L., Green R., Wei Y., Yamaguchi S., Joerger R., Haas M.J., Derewenda Z.S. (1994). Current progress in crystallographic studies of new lipases from filamentous fungi. Protein Eng..

[35] Berg O.G., Cajal Y., Butterfoss G.L., Grey R.L., Alsina M.A., Yu B.Z., Jain M.K. (1998). Interfacial activation of triglyceride lipase from Thermomyces (Humicola) lanuginosa: Kinetic parameters and a basis for control of the lid. Biochemistry.

[36] Fernandez-Lafuente R. (2010). Lipase from Thermomyces lanuginosus: Uses and prospects as an industrial biocatalyst. J. Mol. Catal. B Enzym..

[37] Bastida A., Sabuquillo P., Armisen P., Fernandez-Lafuente R., Huguet J., Guisan J. (1998). A single step purification, immobilization, and hyperactivation of lipases via interfacial adsorption on strongly hydrophobic supports. Biotechnol. Bioeng..

[38] Fernandez-Lafuente R., Armisén P., Sabuquillo P., Fernández-Lorente G., Guisán J.M. (1998). Immobilization of lipases by selective adsorption on hydrophobic supports. Chem. Phys. Lipids.

[39] Bravo Rodríguez V., Jurado Alameda E., Martínez Gallegos J.F., Reyes Requena A., García López A.I. (2006). Enzymatic hydrolysis of soluble starch with an alpha-amylase from Bacillus licheniformis. Biotechnol. Prog..

[40] Shewale S.D., Pandit A.B. (2007). Hydrolysis of soluble starch using Bacillus licheniformis alpha-amylase immobilized on superporous CELBEADS. Carbohydr. Res..

[41] Pandey A., Nigam P., Soccol C.R., Soccol V.T., Singh D., Mohan R. (2000). Advances in microbial amylases. Biotechnol. Appl. Biochem..

[42] Riley R.G., Zachara J.M., Wobber F.J. (1992). Chemical Contaminants on DOE Lands and Selection of Contaminant Mixtures for Subsurface Science Research.

[43] Jeyaratnam J. (1990). Acute pesticide poisoning: A major global health problem. World Health Stat. Q..

[44] Isbister G.K., Mills K., Friberg L.E., Hodge M., O’Connor E., Patel R., Abeyewardene M., Eddleston M. (2007). Human methyl parathion poisoning. Clin. Toxicol. (Phila).

[45] Von Itzstein M. (2007). The war against influenza: Discovery and development of sialidase inhibitors. Nat. Rev. Drug Discov..

[46] Rodríguez-Aparicio L.B., Ferrero M.A., Reglero A. (1995). N-acetyl-d-neuraminic acid synthesis in Escherichia coli K1 occurs through condensation of N-acetyl-d-mannosamine and pyruvate. Biochem. J..

[47] Luchansky S.J., Yarema K.J., Takahashi S., Bertozzi C.R. (2003). GlcNAc 2-epimerase can serve a catabolic role in sialic acid metabolism. J. Biol. Chem..

[48] Ball J.C., Puckett L.G., Bachas L.G. (2003). Covalent immobilization of beta-galactosidase onto a gold-coated magnetoelastic transducer via a self-assembled monolayer: Toward a magnetoelastic biosensor. Anal. Chem..

[49] Hu S., Chen J., Yang Z., Shao L., Bai H., Luo J., Jiang W., Yang Y. (2010). Coupled bioconversion for preparation of *N*-acetyl-d-neuraminic acid using immobilized *N*-acetyl-d-glucosamine-2-epimerase and *N*-acetyl-d-neuraminic acid lyase. Appl. Microbiol. Biotechnol..

[50] Wang T.-H., Chen Y.-Y., Pan H.-H., Wang F.-P., Cheng C.-H., Lee W.-C. (2009). Production of N-acetyl-D-neuraminic acid using two sequential enzymes overexpressed as double-tagged fusion proteins. BMC Biotechnol..

[51] Venning-Slater M., Hooks D.O., Rehm B.H.A. (2014). *In vivo* self-assembly of stable green fluorescent protein fusion particles and their uses in enzyme immobilization. Appl. Environ. Microbiol..

[52] Jahns A.C., Rehm B.H.A. (2009). Tolerance of the Ralstonia eutropha class I polyhydroxyalkanoate synthase for translational fusions to its C terminus reveals a new mode of functional display. Appl. Environ. Microbiol..

[53] Pescador P., Katakis I., Toca-Herrera J.L., Donath E. (2008). Efficiency of a bienzyme sequential reaction system immobilized on polyelectrolyte multilayer-coated colloids. Langmuir.

[54] Aranaz I., Ramos V., de La Escalera S., Heras A. (2003). Co-immobilization of d-hydantoinase and d-carboamylase on Chitin: Application to the Synthesis of p-hydroxyphenylglycine. Biocatal. Biotransform..

[55] Crestini C., Melone F., Saladino R. (2011). Novel multienzyme oxidative biocatalyst for lignin bioprocessing. Bioorg. Med. Chem..

[56] García-Fruitós E., Vázquez E., Díez-Gil C., Corchero J.L., Seras-Franzoso J., Ratera I., Veciana J., Villaverde A. (2012). Bacterial inclusion bodies: Making gold from waste. Trends Biotechnol..

[57] García-Fruitós E., Sabate R., de Groot N.S., Villaverde A., Ventura S. (2011). Biological role of bacterial inclusion bodies: A model for amyloid aggregation. FEBS J..

[58] Peternel S., Komel R. (2011). Active Protein Aggregates Produced in Escherichia coli. Int. J. Mol. Sci..

[59] Carrió M., González-Montalbán N., Vera A., Villaverde A., Ventura S. (2005). Amyloid-like properties of bacterial inclusion bodies. J. Mol. Biol..

[60] Jahns A.C., Maspolim Y., Chen S., Guthrie J.M., Blackwell L.F., Rehm B.H.A. (2013). *In Vivo* Self-Assembly of Fluorescent Protein Microparticles Displaying Specific Binding Domains. Bioconjug. Chem..

